# The Sources of Research Self-Efficacy in Postgraduate Nursing Students: A Qualitative Study

**DOI:** 10.3390/healthcare10091712

**Published:** 2022-09-07

**Authors:** Jiali Gong, Meizhen Chen, Qiuping Li

**Affiliations:** Wuxi School of Medicine, Jiangnan University, Wuxi 214122, China

**Keywords:** nursing education, postgraduate, qualitative study, research self-efficacy

## Abstract

Postgraduate students are required to undertake a series of research assignments. Research self-efficacy is regarded as the sense of faith in their ability to accomplish research tasks. However, it is unclear how research self-efficacy plays a role in second-year nursing postgraduate students who have been enrolled for a period of time and have not yet met graduation requirements. This study aims to explore the sources of second-year nursing postgraduate students’ research self-efficacy in response to research tasks. A descriptive phenomenological approach was used in this study. A total of 13 second-year nursing postgraduate students participated in semi-structured interviews using a purposive sampling method. Content analysis was applied to analyze the interview data after verbatim transcription. Participants stated that they had gained a great deal of support but were somewhat less confident. Three themes were refined: (a) intrinsic sources of research self-efficacy (differences in cognitive ability, internal driving force, with successful experience), (b) extrinsic sources of research self-efficacy (family support, peer support, mentor support), and (c) unmet support may cause low self-confidence (inadequate self-support, inadequate extrinsic-support, lack of motivation from successful experiences). The postgraduate students in this study accepted the objectives of the master’s training and actively practiced research exploration. They were motivated to persevere through their internal and external support, albeit with some negative aspects that deserve our attention. Interventions for postgraduate students’ cognitive awareness, constant support during their postgraduate career, and finally, guiding summaries may have a positive impact on their self-efficacy.

## 1. Introduction 

In China, nursing postgraduate education started in the 1990s [1,2]. Postgraduate education aims to cultivate high-level nursing professionals for society, who will be engaged in nursing academic research, nursing management, and nursing education in the future [3]. Moreover, studies have revealed that an enhancement of nurses’ educational level is beneficial not only for raising their competence level but also for benefiting patients by improving the level of care [4,5]. Consequently, there is a rising societal demand for high-level nursing professionals. Driven by demand and supported by government policy, an increasing percentage of nursing staff have been applying for master’s programs. With the admission of postgraduate students, they are required to undertake a series of assignments as a research procedure, which refers to the process of going through the identification of a topic, data collection, and data analysis to writing and even publishing a paper.

Assignments emanating from the pursuit of a master’s degree create several predictable pressures on postgraduate nursing students [6]. The pressure generally stems from research tasks, time management, graduation requirements, and the environment of study [7,8]. As the findings show, new postgraduate students fall into negative emotions such as anxiety and depression after enrollment, which could even affect their physical and mental health in severe cases [9]. To gain a master’s degree, postgraduate nursing students need to overcome multiple challenges and the resulting stress and negativity. It is advisable to pay adequate regard to the stress that postgraduate nursing students experience during their master’s studies and to find appropriate approaches to cope with it. 

An essential coping mechanism to overcome challenges until extrinsic interventions arrive is the self-efficacy of nursing postgraduate students, particularly research self-efficacy [10], which refers to the sense of faith in their ability to accomplish a given task [11,12]. This self-efficacy drives postgraduate students to take the initiative to push their research progress and develop individual research plans to be executed in a structured manner. Bandura pioneered the self-efficacy theory, which attributes self-efficacy to the following four main sources: performance accomplishment, vicarious experience, verbal persuasion, and emotional arousal [13]. As the words suggest, self-efficacy is not always acquired in one way; it can be acquired through the accumulation of experience in one’s own behavior or the guidance of others, and positive emotions can also enhance postgraduate students’ self-efficacy, which has the attributes of being personal, malleable, goal-driven, resourceful, knowledgeable, and trustworthy. Self-efficacy in postgraduate students fosters constructive ideas, feelings, and behaviors that lead to positive outcomes [14]. They may be motivated to complete postgraduate studies and earn a master’s degree as long as their research self-efficacy can be improved through a supportive program setting.

To successfully structure a support program that enhances the research self-efficacy of postgraduate nursing students, it is necessary to comprehend what their research self-efficacy stems from so that it meets their expectations and is acceptable to them. Presently, studies have proposed support for first-year postgraduate students preparing to enroll, such as in transition courses, to accelerate their adjustment to the pace of postgraduate study [15]. However, few document support for second-year postgraduate students, those who have been enrolled for a period of time, and have not yet met graduation requirements.

To summarize, the purpose of this study was to explore the experiences of second-year nursing postgraduate students’ research self-efficacy in response to research tasks during their master’s degree studies through a qualitative research approach. According to their real experiences, we can distill the current unmet needs and provide a rationale for designing future support programs to improve their self-efficacy.

## 2. Method

### 2.1. Study Design

This is a qualitative phenomenological study based on interview data to identify the sources of research self-efficacy of nursing second-year postgraduate students during their master’s studies. The findings of this study were synthesized using the Consolidated Criteria for Reporting Qualitative Research (COREQ) [16].

### 2.2. Participants

We extended an invitation to eligible second-year postgraduate students in nursing studying at a university in Jiangsu Province, China. The inclusion criteria were (a) they are second-year postgraduate nursing students; (b) their type of education is full-time or part-time, and (c) they would be cooperative and were capable of good communication. Participants were selected by using the purposive sampling method and recruited to continue until data saturation was reached when no new themes were emerging.

When participants were found to be eligible, J.G., a postgraduate nursing student, approached them by telephone in a timely manner to present them with verbal and written information about the study. Those who were interested in participating in the study were asked to sign a formal agreement for participation and return it to the researchers. When the 13th participant was reached, the data had reached saturation, at which time the first and second authors jointly confirmed that no new themes or subthemes had emerged. The mean age of participants was 26 years (range 22–45 years). The demographic information of the participants is shown in Table 1. 

### 2.3. Data Collection

From January 2022 to February 2022, data were gathered through semi-structured interviews in a college or hospital setting. The time and location of the interviews were selected by the participants. J.G. and M.C., who are postgraduate nursing students with expertise in conducting qualitative research interviews, performed the interviews. The interviews were taped, and notes were collected throughout interviews to capture participants’ body language and facial expressions. Only the participant and the researcher were present for each interview. We used WeChat video or voice conferencing whenever face-to-face interviews were not possible due to the COVID-19 pandemic.

The interview guide was jointly developed through team group discussion based on the related literature review. The group members included master’s degree students and supervisors. They were familiar with research self-efficacy. Prior to the team group discussion, we reviewed the literature on self-efficacy together and gained an understanding of what self-efficacy means, which is a faith in one’s ability to accomplish a task in a specific domain [13,14]. The first question was identified according to the specific tasks of self-efficacy in the field of research, including data collection, analysis, research integration, paper writing, etc. To address these tasks, we asked how the participants deal with these research tasks and what were the sources of their confidence in the process of coping with research tasks. In this regard, we decided to set questions 2 and 3. According to the study, it is known that emotions can influence an individual’s self-efficacy. We have yet to understand which moments make students feel proud or feel confident. To help find directions for future supportive practice, we considered setting question 4. The consists of a list of open-ended questions used in the semi-structured interview. To evaluate the semi-structured interview questions and teach the researchers, two pilot interviews were conducted. During the pilot interviews, participants frequently mentioned that they felt pride even after being frustrated, which led to a small modification by adding question 5 for understanding the moment of frustration. The interview guide is presented below (Table 2). A total of 13 face-to-face interviews and three telephone interviews were conducted. The three telephone interviews were conducted as a second interview with three participants for the reason of additional interview content to ensure interview completeness. The mean length of the interviews was 27.1 min (range, 13–65 min).

### 2.4. Data Analysis

We used the content analysis method of qualitative research [17]: (a) The first author (J.G.) transcribed the recorded interview data verbatim and imported the text into a computer-aided qualitative data analysis software (CAQDAS), NVivo software (version 12.0, QSR International, Doncaster, Victoria, Australia). The transcripts were given feedback by participants and compared with the audio to ensure accuracy; (b) the text was broken down into units of meaning by multiple close readings and then condensed to make it shorter without sacrificing any important information. These meaning units were given a code; (c) codes were recombined to find themes and subthemes based on the differences and similarities; (d) two authors (J.G. and M.C.) cross-reviewed the themes; (e) when disagreements occurred, the author team discussed them to resolve issues and develop a final theme and sub-themes, and (f) finally, the final report was produced by J.G.

### 2.5. Rigor

The findings’ credibility was ensured by applying Lincoln and Guba’s guidelines [18]. Peer debriefing and member checks helped establish credibility. To assure the accuracy of the recorded data, specialists checked the substance of the transcript and participants confirmed it. Moreover, to verify that the recorded data were accurate, all authors double-checked the data after each interview, and their opinions were incorporated into the final analysis.

## 3. Results

A total of 71 codes were initially identified. These were reduced to 46 after merging duplicates or similar codes. These codes were classified according to their commonalities after thorough and iterative procedures. This final step made it possible to create three themes: (a) intrinsic sources of research self-efficacy, (b) extrinsic sources of research self-efficacy, and (c) unmet support may cause low self-confidence. The three themes included a total of nine sub-themes. (Table 3).

### 3.1. Intrinsic Sources of Research Self-Efficacy

The intrinsic source of self-efficacy exists within the participants themselves, who recognize research tasks as a challenge that could be surmounted, are highly motivated to take action as well as proactive in taking stock and reflecting after completion. This theme could be reduced to three sub-themes as follows:

#### 3.1.1. Differences in Cognitive Ability 

Some participants perceived that life is a process and focused on what they can experience rather than caring about where the end is. Thus, they were willing to try some challenging tasks:

*“Individuals may feel comfortable when they are in their comfort zone, but I think human life is limited. We should do what we like or what we want to challenge, only then will we not have any regrets. That’s why I insisted on choosing to pursue a master’s degree.”*

(P11)

When participants encountered difficulties, they were able to view it as motivation and push themselves to devote their energy to the research:

*“I felt that it was difficult when I was applying for my master’s subject. I didn’t do anything special at the time; I just went to the library and read the literature carefully. I pushed myself to complete the task no matter what.”*

(P7)

Participants indicated that they would reflect on the study when it was completed, including the meanings of the study and how they performed during the study. This reflection urged them to modify their methods or rectify previous deficiencies the next time:

*“As I analyze the data, I think about what the implications of doing so are. Is it what I think it is? Does it actually mean the same thing as I personally and subjectively think it means?”*

(P11)

#### 3.1.2. Internal Driving Force

Participants stated that they felt more confident in completing the task when they viewed the research task as a challenge that they could overcome while maintaining the self-discipline they had previously developed in terms of learning. When faced with research challenges, they reported upbeat emotional arousal:

*“I am confident in my own abilities and have also developed the habit of independent study. I feel that I can complete my research.”*

(P9)

If they faced difficulties in their research, participants indicated that they would take the initiative to consult seniors to facilitate their research:

*“I used to follow along as my seniors did their experiments and I would ask them to teach me, if needed, e.g., ‘can you tell me how to do this’. That’s how I feel my way around and if I fail, then I do it again.”*

(P8)

Once the daily scheduled tasks were completed, participants reported that they felt a sense of fulfillment and satisfaction, and they enjoyed this sense of gratification:

*“I feel uneasy when I waste precious time. I prefer to be in a busy state, which is very fulfilling and I like that. There’s a sense of achievement when I’ve completed all the work, “I’ve finally done it!” It’s very fulfilling.”*

(P7)

In addition, they desired to capture the spirit of conscientiousness, self-discipline, and exploration as a way to encourage those around them and become better with them:

*“When I was preparing for my postgraduate entrance exams and my son was in high school. I felt that I should also study so that we could promote each other instead of relaxing at home watching TV shows. Although most people in my age don’t continue their studies anymore, I do not agree with their attitude to life.”*

(P11)

#### 3.1.3. With Successful Experience

Research work started with reading the literature. Some participants realized that they can effectively capture information from the literature right from the start, which is a bit insignificant, but they were nonetheless happy about it:

*“What I do best is to distill the information from the literature. After reading the literature, I would sort out a general idea of what the piece of literature was about. So, I am great at capturing information.”*

(P7)

Debriefing in group meetings on what they have learned from reading the literature required overcoming mental barriers and connecting knowledge logically rather than reading more and more literature. They often summarized methods from previous debriefing experiences:

*“The most impressive thing for me was presentation at the group meeting. I had to spend a long time every week reading literature, and I had to try to make others understand me when I presented at the group meeting, which was also a kind of ability.”*

(P8)

This incremental success encouraged participants to venture into uncharted territories, such as data collection, which they had not studied as undergraduates. Participants stated that they associated other prior experiences to help them accomplish somewhat similar tasks:

*“I do believe I am capable of achieving the completion of the research data collection. After all, I have five years of clinical work experience. If I really want to do a nursing project, I’m sure I can do it.”*

(P13)

Even without prior experience, participants expressed confidence that they could complete the tasks if they started study at the postgraduate stage:

*“I am confident in data analysis as I have studied it before, so, I am familiar with it. Because I need to analyze data with my group members, I have learned how to analyze data since I started my master’s degree study.”*

(P10)

### 3.2. Extrinsic Sources of Research Self-Efficacy

While the efforts of postgraduate students themselves are important, extrinsic supports are also essential factors in stimulating their dedication to research. This support not only provides direct assistance to the participants but also serves as a model for them to follow. They frequently commented on three main sources of extrinsic support: family, peers, and mentors. Therefore, this theme was reduced to three sub-themes as follows:

#### 3.2.1. Family Support

Participants who received family support indicated that their relatives had high expectations and exigent demands on them, which were reflected in scientific achievements, future employment, etc.:

*“My family has relatively high expectations of me and wants me to obtain higher qualifications. They hope that I will be able to hold a decent job and earn a good income, that I will have better living conditions and be able to live a happy and fulfilling life.”*

(P2)

At the same time, the family also provided assistance such as financial support, which improved the participant’s standard of living and helped her to devote herself to research:

*“They gave me financial support so that I didn’t have to worry about living during my postgraduate studies.”*

(P9)

Other participants reported that family support consisted of more of emotional support, ss such kindness, love, encouragement, and understanding. Family members did not concern themselves with how successful the participants were but more about whether they were comfortable and happy in life:

*“My parents always encourage me to do my research. Well, they love me a lot and they are very supportive whether I am good or not. Of course, with the encouragement of them, I have more confidence in myself.”*

(P6)

#### 3.2.2. Peer Support

Participants stated that they always spent time with their roommates or study group members after enrollment. Peers would help each other with their research, and this help was more specific for tangible research tasks:

*“I learned a lot of knowledge and skills from my roommates. For example, when searching for literature, she would say “this place would be better if you used another method to search for it, and it would be better if you put a plug-in on your browser.”*

(P7)

Hence, peers were more likely to have common topics of conversation with each other. Participants indicated that they would share their life experiences with their peers or vent negative emotions that arose during the research process:

*“We are always together and keep each other company. When the mood strikes, we go out for a walk together and enjoy a good meal to distract ourselves. We vent out our worries to each other to reduce stress. I feel good about that.”*

(P5)

Furthermore, participants also perceived that this feeling of being supported was stronger than ever when they were part of a friendly and harmonious team. Such peer support as shown above primarily provided participants with an unhindered access to **verbal persuasion** with accessibility and portability:

*“The members of my subject group are all very nice and we get along very well. For example, when I forget to have lunch due to involving in my work, my partner will buy the lunch and bring it to me in my office. This relationship really sustains us all.”*

(P13)

#### 3.2.3. Mentor Support

Participants would apply for a mentor to guide them after enrollment, who would be a facilitator in their research process. Such mentorship is reflected in the daily communication with the mentor. Participants reported that the mentor formed a friendly relationship with them as an equal communicator, which usually led to inspiration for them:

*“My mentor is more casual in his conversations with me and he’s usually not very serious. He always listens to me after he has given his opinion. This kind of listening makes me more willing to express my ideas.”*

(P3)

Participants reported that their mentors gave them appropriate feedback during their time together, which was mainly guidance in their areas of expertise. Mentors usually have a wealth of experience in research and constitute the most professional **vicarious experience** that participants deserved to learn from:

*“I frequently discuss my subject with my mentor, who provides me with helpful advice that pushes me ahead. A word from my mentor can wake me up.”*

(P10)

Mentor support is not merely a passive provision of experience, but they can act as a monitor or facilitator in the research process. Participants reflected that their mentors not only provided guidance but also pushed their research progress with pressure at appropriate times, which was well-suited to mitigate the inefficiencies associated with occasional delays:

*“My mentor invites me to his office every week to talk to him. Even though the progress of my subject has been slow lately, he is still willing to communicate with me. I appreciate this way of spending time together.”*

(P3)

### 3.3. Unmet Support May Cause Low Self-Confidence

However, it was possible that when support was not appropriate, the experience was not developed, resulting in a lack of self-confidence. Some participants described feelings of unfulfillment as a constant during the year, which caused them to be potentially less motivated for research tasks. This theme could be reduced to three sub-themes as follows: 

#### 3.3.1. Inadequate Self-Support

In terms of intrinsic support, participants expressed their feelings of unfulfillment stemming from a lack of interest in the research. Thus, it was hardly possible for participants to evoke positive coping emotions regarding research:

*“It’s mainly because I don’t love research enough. The main reason for choosing nursing as the major is the great number of jobs available, and it is relatively easy to find a job.”*

(P2)

When not fully engaged in the research, participants indicated that the ability to develop a sound research plan was correspondingly diminished, as engagement might be regarded as a process that is affected by the antecedents (e.g., learning interest) and affects the consequences (e.g., research schedule):

*“Since I spent most of my time selecting the project in the first phase, and I was afraid that there was also a lot of work in the data collection, which might lead to not enough time for the analysis later. So, I became more worried that I wouldn’t be able to finish as scheduled.”*

(P1)

Time was underutilized when participants were stuck at a certain step in the research. Participants seemed more likely to give themselves negative ratings, which were reflected in a lack of confidence in their own research abilities:

*“I think I am poor at summarizing. Let’s say I was asked to summarize the content of 20 pieces of literature. I think it would be very difficult. I even talked to my mentor about this in my meeting with him yesterday.”*

(P3)

#### 3.3.2. Inadequate Extrinsic Support

A decrease in intrinsic support leads to a decrease in extrinsic support for postgraduate students who are primarily self-directed learners. A part of the extrinsic support stems from the initiative that the participants pursued, and while there is a lack of initiative from the participants, the strength of extrinsic support is inevitably weakened:

*“My supervisor is not from a nursing background and I lack the guidance of corresponding nursing mentors in studies. So, I feel that I am not very confident in my research.”*

(P4)

On the one hand, they believed that the lack of extrinsic support was the main cause of the slow progress of their research. On the other hand, they also ignored self-support. However, it is undeniable that the lack of extrinsic support invites dissatisfaction from the participants:

*“My mentor only had me as a student, as my mentor had also just started enrolling, so I had no help from the research group and no seniors to show me the ropes.”*

(P12)

#### 3.3.3. Lack of Motivation from Successful Experiences

Interviews showed that participants were intimidated to do research even if extrinsic assistance was available. With no previous experience of success, they were unsure whether the support would really work for them:

*“The next step was data collection from my subject. Although the head nurse said she would help me, I was afraid to take that step. Because there are still very few exercise interventions in the country, I was afraid that if I did carry out one, it would not be well received.”*

(P3)

While both successes and failures prompted participants to reflect and progress in some way, failures were more likely to induce negative emotions that could hinder the research:

*“I think I’m pretty bad at it. I haven’t been good at writing since I failed in language as a child, so I think I might have a little bit of difficulty with the essay writing piece caused of my previous failures.”*

(P10)

There was a correlation between the three themes. Intrinsic support can facilitate the absorption of extrinsic support. Intrinsic support is a self-driven force that assists participants in applying the knowledge and skills they already have and in seeking external support such as experience (**vicarious experience**) and guidance (**verbal persuasion**) in time to transform them into the raw materials needed to tackle the research task when capacity is lacking. Intrinsic support will also help them transform these experiences into **performance experiences** after completing the challenge. While both success and failure can be viewed as performance experiences, successful experiences also help participants **arouse positive emotions**, which are as important to their sense of self-efficacy as intrinsic/extrinsic motivation. However, some participants lacked confidence in the research. Not only did they lack intrinsic support, but they also had difficulty in obtaining effective external support, resulting in a lack of accumulation of successful experiences. 

## 4. Discussion 

Nursing second-year postgraduate students’ sources of research self-efficacy in coping with research assignments throughout their master’s degree studies were elicited through interviews. It was observed that students who are intrinsically motivated and adequately supported tend to perform self-efficacy well in their research tasks. A total of three themes were identified in this study: intrinsic sources of research self-efficacy, extrinsic sources of research self-efficacy, and unmet support may cause low self-confidence.

As found by interviews, there were three personal idiosyncratic features reflected in the attitudes of some participants who viewed challenges. To begin with, the more confident individuals expressed that they preferred the experience of dealing with the difficulty to the outcome (subtheme “differences in cognitive ability” under “intrinsic sources of research self-efficacy”). Although they encountered difficulties, the emotions of positive response to difficulties were evoked. Unlike the participants in this study, other second-year postgraduate students in medical programs believed that professional learning was difficult and showed concern about the training and the work they would face in the future [19]. This gave rise to negative psychological states such as anxiety and depression. However, there are differences between programs, and this finding should be taken with caution. The participants in the present study pursued their master’s degree with goals, and they had a high level of self-efficacy while being able to focus on the experience, which is in line with the previous study [20,21]. First-year postgraduate students also agree that it is beneficial to have clear expectations at the beginning [22]. The initial goal in first year is probably broad, such as promoting skills and gaining knowledge. The goal was refined in the second year, where students had clear goals for addressing their research tasks. Two other personal features are the capacity to focus and active reflective behavior, which are traits that match well with self-efficacy [12]. As a consequence, these traits develop externalized behaviors, including actively seeking support, positively evaluating oneself, and allowing one’s energy to influence others around them (subtheme “internal driving force” under “intrinsic sources of research self-efficacy”). This reflective behavior was also reflected in first-year postgraduate nursing students who took the initiative to uncover aspects of similar previous experiences that would be useful for subsequent research studies [15]. Cognitive interventions could be one approach for postgraduate students to regain a handle on these personal traits [23]. In the meantime, we need to focus more on time spots (subtheme “with successful experience” under “intrinsic sources of research self-efficacy”). The first successful moment is important for postgraduate students because it may motivate them to continue the research process, and the moment of the end of a study, which prompts them to take stock of the experience in time, is also beneficial for them to start a new task or complete a parallel task of the same type. While first-year nursing postgraduate students and other program graduate students agree on the importance of performance experiences, second-year postgraduate students seem to place emphasis on the first successful moment in graduate study [15,24].

Postgraduate students are regarded as autonomous and independent, whereas they still need help and support to develop capability, which promotes the accumulation of knowledge and skills that later enhance self-efficacy [25]. The second-year nursing postgraduates in the present study reported receiving families support, which was motivated by love, the power of kinship, and the presence of family expectations for the postgraduates that were essentially aligned with their goals. As a result, participants stated that their family duties and student roles were well-balanced, which is beneficial to self-efficacy improvement [26] (subtheme “family support” under “extrinsic sources of research self-efficacy”). Family support is also a concern for first-year postgraduate students, as they are concerned about the financial burden of enrolling in school, which can be somewhat alleviated with the support of their families [27]. Participants in this study also affirmed that peer support was a guarantee that they would be more confident as well as positive in addition to the support from peers being more specific and closer than that from family members or mentors [28,29] (subtheme “peer support” under “extrinsic sources of research self-efficacy”). The role of peer support was also recognized by preparing for admission of postgraduate nursing students as promoting personal and professional development [28]. Peer support was repeatedly mentioned in the expressions of second-year postgraduate students, who seemed to feel more strongly about it. Being together for a long period gave them an emotional dependence to comfort and encourage each other. Another finding of this study was that the participants, together with their peers, constituted a cordial, reciprocal, and harmonious academic environment, where they became more confident in their capacity to overcome academic challenges [30,31]. Nursing students from other grades also affirmed the importance of peer involvement in collaborating to reach goals as well as the importance of the learning environment [32]. However, when the environment is unstable, the impact will be greater for second-year than for first-year postgraduate students [33]. Therefore, creating a stable academic environment is crucial for second-year students.

Students may have natural feelings of respect and admiration for their mentors. They place a strong value on the interactive contact between mentors and themselves [34]. Being with the mentor is unique because peer support usually involves multiple members that are substitutable, whereas mentors are usually definite and unchangeable; consistent with previous research, postgraduate students in this study expressed a greater expectation to engage in dialogue with their mentors on an equal footing, like a collegial interaction [35]. It is effective communication that will alleviate misunderstanding and disconnect between each other [36]. Findings on first-year nursing postgraduates and other medical postgraduates also showed that graduate students were more likely to achieve higher performance when they received high levels of support from their mentors [15,37]. First-year postgraduates expect mentors to take a leadership role, while second-year postgraduate students seem to be more willing to have continuous interaction and communication with their mentors. The delay of information and the lack of timely feedback may come out when they fail to form a well-developed relationship, undermining the self-efficacy of postgraduate students [38]. It is also beneficial for mentors to maintain a well-developed relationship with postgraduate students in the dimension of mentor. Otherwise, there is a risk of increasing accusations for mentors who will suffer from pain and uneasiness and thus lose enthusiasm for mentoring students [39].

To sum up, the second-year nursing postgraduates showed a positive attitude towards the difficulties they faced, and they gradually set clear goals. The first successful research experience motivated them to keep going. In terms of external support, they expect to have a relatively stable academic environment and deeper interaction with people around them (family, peers, and mentors). 

### 4.1. Practical Implication

The following is an attempt to summarize the future practice of self-efficacy support for second-year postgraduate nursing students through the four sources of self-efficacy.

#### 4.1.1. Performance Experiences

Performance experience helps second-year postgraduate students to accumulate success experiences and change their attitudes towards difficulties. Reliance is placed on pre-existing experiences before enrollment and the attitudes of postgraduate students as they view the situation. Cognitive interventions were recommended as the main approach to support in this area [40].

#### 4.1.2. Vicarious Experience

Peer support to create an academic environment and mentor support to provide professional guidance is recommended in this area. Effective vicarious experiences can be facilitated in a positive academic environment. Peer support can be delivered in groups or one-on-one [41]. Establishing a supportive relationship during postgraduate studies may also facilitate a collaborative relationship later on [42].

#### 4.1.3. Verbal Persuasion

Students should encourage mutual assistance with those around them regardless of the role of the supporter. Stakeholders involved in the education of master’s students in nursing (postgraduate students, mentors, parents, managers, etc.) need to raise awareness in this area. Actions such as organizing counseling sessions for postgraduates to provide professional guidance would benefit master’s students research. Stakeholders’ thoughts should be sought in this regard in the future.

#### 4.1.4. Emotional Arousal

Positive emotions enhance their confidence in coping with research tasks. The pleasure of success also makes people want to achieve success repeatedly, with positive emotions coming from success in research challenges. Helping postgraduate students deconstruct large research tasks and accumulate more successes may be one way to feel more optimistic. Actions such as offering an elective course in positive psychology would facilitate students to learn to self-regulate and to obtain positive emotions such as satisfaction and happiness in research practice [43].

### 4.2. Study Limitations

There are some limitations to this study. The study was conducted in female participants due to the few men who enrolled in postgraduate. Future qualitative research on the research self-efficacy of male nursing postgraduate students could be added. In addition, the small sample size and participants’ different life experiences made it impossible to determine the degree of each influencing factor. Future research could further explore this aspect in depth. Moreover, since all the samples were from a university located in Jiangsu Province, the influence of the region on the results cannot be excluded. Future studies can be conducted in other provinces and regions.

## 5. Conclusions

The postgraduate students in this study accepted the objectives of the master’s training and actively practiced research exploration. They were motivated to persevere through their self- and external support, albeit with some negative aspects that deserve our attention. Early intervention for postgraduate students’ cognitive awareness, constant support during their postgraduate career, and, finally, guiding summaries may have a positive impact on their self-efficacy.

## Figures and Tables

**Table 1:** Characteristic of nursing postgraduate students.

Number	Age (y)	Gender	Job Title	Marital Status	Enrollment Pathway	Education before Graduate School	Work Tenure	Type of Master’s Study
P1	25	Female	No	Unmarried	General entrance examination	Bachelor’s Degree	Two months	Full-time
P2	23	Female	No	Unmarried	General entrance examination	Bachelor’s Degree	No	Full-time
P3	24	Female	No	Unmarried	General entrance examination	Bachelor’s Degree	No	Full-time
P4	24	Female	No	Unmarried	General entrance examination	Bachelor’s Degree	No	Full-time
P5	24	Female	No	Unmarried	General entrance examination	Bachelor’s Degree	No	Full-time
P6	22	Female	No	Unmarried	Exemption entrance examination	Bachelor’s Degree	No	Full-time
P7	27	Female	No	Unmarried	General entrance examination	Bachelor’s Degree	2.5 years	Full-time
P8	24	Female	No	Unmarried	General entrance examination	Bachelor’s Degree	No	Full-time
P9	23	Female	No	Unmarried	Exemption entrance examination	Bachelor’s Degree	No	Full-time
P10	25	Female	No	Unmarried	General entrance examination	Bachelor’s Degree	No	Full-time
P11	45	Female	Vice senior	Married	General entrance examination	Collage	25 years	Part-time
P12	24	Female	No	Unmarried	General entrance examination	Bachelor’s Degree	1 year	Full-time
P13	28	Female	Primary	Unmarried	General entrance examination	Bachelor’s Degree	5 years	Full-time

**Table 2:** Interview guide for nursing postgraduate students.

Number	Questions
1	Are you confident that you are up to the task of research? Please share it with us based on your experience (data collection, analysis, research integration, paper writing, etc.)
2	How did you usually deal with research pressure or problems during your master’s degree?
3	What aspects of support/motivation have led you to be more confident in your research tasks?
4	Tell us about something that made you proud during your master’s degree.
5	Tell us about something that frustrated you during your master’s degree.

**Table 3:** Themes and sub-themes.

Themes	Sub-Themes
Intrinsic sources of research self-efficacy	Differences in cognitive ability
	Internal driving force
	With successful experience
Extrinsic sources of research self-efficacy	Family support
	Peer support
	Mentor support
Unmet support may cause low self-confidence	Inadequate self-support
	Inadequate extrinsic-support
	Lack of motivation from successful experiences

## Data Availability

We (the authors) have full control of all primary data and agree to allow the journal to review the data if requested.
